# Ultrasound biomicroscopic imaging analysis of lens position and stability in acute and chronic angle-closure glaucoma

**DOI:** 10.3389/fopht.2025.1624876

**Published:** 2025-07-02

**Authors:** Zhiying Yu, Xinyu Wang, Haitao Wang, Jing Han, Jing Fu, Licun Wang, Ling Wang

**Affiliations:** ^1^ Department of Ophthalmology, The Affiliated Hospital of Qingdao University, Qingdao, China; ^2^ Department of Security and Logistics, The Affiliated Hospital of Qingdao University, Qingdao, China

**Keywords:** acute angle-closure glaucoma, chronic angle-closure glaucoma, ultrasound biomicroscopy, lens position, suspensory ligament

## Abstract

**Introduction:**

This study aimed to compare the characteristics and differences in lens position and stability in patients with acute and chronic angle-closure glaucoma (ACG) using ultrasound biomicroscopy (UBM) to provide a basis for selecting treatment regimens for primary ACG (PACG).

**Methods:**

This prospective study included 82 eyes of patients with PACG, of which, 45 eyes with acute PACG (APACG), 37 with chronic PACG (CPACG). Axial length (AL) and lens thickness (LT) were measured using A-scan ultrasonography. Anterior chamber depth (ACD), pupil diameter (PD), and lens vault (LV) were measured using UBM for each group. Additionally, trabecular-iris angle (TIA), angle opening distance (AOD_500_), iris-lens angle (ILA), and iris-lens contact distance (ILCD) were measured in four quadrants (superior, inferior, nasal, and temporal) with UBM. The corresponding lens position (LP), relative lens position (RLP), and lens thickness/axial length factor (LAF) were calculated. Normally distributed data were compared between the two groups using an independent samples t-test. Data that did not follow a normal distribution were compared using the Mann–Whitney U test. Differences were considered statistically significant when *P* < 0.05, and they were considered highly statistically significant when *P* < 0.01.

**Results:**

The values for angle-related parameters, including the mean TIA, TIA_max-min_, mean AOD_500_, AOD_500 max-min_, and ACD, were significantly lower in the APACG group than in the CPACG group (all *P* < 0.05). The LP and RLP values of the APACG group were also lower than those of the CPACG group, but only the difference in LP values being statistically significant (*P* = 0.038). The LT, LV, LAF, mean ILCD, and ILCD_max-min_ values were higher than those of the CPACG group, with the differences reaching statistical significance (all *P* < 0.05).

**Conclusion:**

The APACG eyes had a thicker and more-anteriorly positioned lens than those with CPACG, which results in a shallower anterior chamber and narrower anterior chamber angle. In the APACG group, the lens exhibited nonuniform laxity of the suspensory ligament across the various quadrants, poor stability, and greater susceptibility for anterior displacement or even deviation.

## 1 Introduction

Glaucoma is a disease that is mainly responsible for visual impairment and irreversible blindness worldwide. Song et al. estimated that, with the acceleration of population aging, the total number of glaucoma cases in China will reach 25.16 million by 2050 (1). Among the various forms of glaucoma, primary angle-closure glaucoma (PACG) has the highest incidence in China and is associated with a higher probability of blindness (2, 3). Therefore, investigating the pathogenesis of PACG and prevention of the disease remains an arduous and crucial task.

Pupillary block is an important pathogenic mechanism of PACG that is mainly affected by lens-related factors, iris-related factors, pupillary dilation, and neurovascular factors. In most cases, PACG is treated by relieving the pupillary block through lens extraction or prophylactic laser peripheral iridotomy (LPI). Certain researchers have suggested that early lens extraction is an effective initial approach for PACG treatment (4). Ong et al. evaluated the effects of lens extraction and other interventional measures for the treatment of chronic PACG (CPACG) and demonstrated that lens extraction was more advantageous than LPI in the treatment of CPACG at the 3-year follow-up visit (5). However, a study by Song et al. that involved the post-lens extraction follow-up of PACG patients revealed that glaucoma progressed postoperatively despite the intraocular pressure (IOP) decreasing (6). The results described above clearly demonstrate the essential role of lens-related factors in the pathogenesis of PACG. However, there is a lack of systematic research assessing whether they serve a similar role in the onset and progression of acute PACG (APACG) and CPACG or whether differences exist in the structure, position, and stability of the lens between these two forms of glaucoma.

Anterior segment optical coherence tomography (AS-OCT) and ultrasound biomicroscopy (UBM) are both important methods for examining the anterior chamber angle. AS-OCT, which has advantages of being non-invasiveness, non-contact, highly safe, and independent from the influence of corneal opacity, has become increasingly popular among examiners (7). However, when used as a tool for optical examination, it provides poor visualization of structures posterior to the iris due to influences of the iris pigment epithelium and refractive media. In contrast, UBM involves a relatively complicated operation and requires a water bath environment but serves as a traditional acoustic examination method that is unaffected by refractive media. It allows for a clear visualization of various tissues and structures of the anterior eye segment in a high-resolution and dynamic manner and facilitates the quantitative measurement of the relevant parameters (8). Our previous study revealed that, despite the greater convenience of AS-OCT, UBM imaging was more advantageous in the measurement of the iris-lens angle (ILA) (9). Therefore, we performed UBM for the analysis of the imaging characteristics and differences in lens position and stability between the APACG and CPACG patients to provide a basis for treatment regimens for these two forms of glaucoma.

## 2 Methods

### 2.1 Study design and patients

This study used a prospective design. Eighty-two PACG patients (82 eyes) admitted to the ophthalmology department of our hospital between July 2023 and October 2024 were selected for the study. Forty-five of the patients (45 eyes) had APACG attacks, and 37 patients (37 eyes) had CPACG. The patients consisted of 29 men and 53 women aged 40–82 years (mean age: 65.46 ± 7.55 y) (**Table 1**).

**Table 1:** General information on the patients.

Variable	APACG (n=45)	CPACG (n=37)	Totel (n=82)	*P*
Mean age, years	65.82 ± 6.26	65.03 ± 8.95	65.46 ± 7.55	0.638^1^
male/female	12/33	17/20	29/53	0.069^2^
IOP (M (P_25_,P_75_)) (mmHg)	21.80 (14.65,50.00)	21.00 (17.00,27.80)	21.05 (15.00,41.68)	0.551^3^
PD (mm)	3.804 ± 1.044	3.371 ± 0.804	3.609 ± 0.962	0.042^1^

^1^independent samples t-test; ^2^chi-square test; ^3^Mann–Whitney U test.

APACG, acute primary angle-closure glaucoma; CPACG, chronic primary angle-closure glaucoma; IOP, intraocular pressure; PD, pupil diameter.

The inclusion criteria were as follows: (1) Patients aged ≥40 years who fulfilled the diagnostic criteria for PACG; (2) unilateral acute attack of APACG with an onset time of <10 days that was poorly controlled by medications (only partially controlled IOP), and had no indications for LPI, and required surgery; (3) CPACG that was poorly controlled by medications and required surgery. Exclusion criteria were as follows: (1) a history of ocular surgery, trauma, or LPI; (2) major underlying diseases that required medical or surgical interventions; (3) patients with limited mobility, inability to adopt a supine position, inability to cooperate, or allergies to anesthetic agents; (4) high myopia with an axial length (AL) of ≥27 mm; (5) secondary glaucoma or ophthalmologic conditions affecting the anterior chamber angle, such as lens subluxation, angle recession, iridodialysis, and occupying lesions in the anterior or posterior eye segments. The study complied with the requirements of the ethics committee of the Affiliated Hospital of Qingdao University and was conducted in accordance with the principles of the Declaration of Helsinki.

**Table 1** shows that age and gender were not significantly different between the two groups (*P* > 0.05). The IOP of the APACG patients was higher than that of the CPACG patients, but the difference was not statistically significant (*P* > 0.05). The pupil diameter (PD) was significantly larger than that of the CPACG group, with the difference being statistically significant (*P* < 0.05).

### 2.2 Research methods

All patients underwent eye examinations one day before surgery (APACG eyes were examined after partial IOP control). Visual acuity examination, slit-lamp microscopy, IOP measurement, computerized optometric examination, gonioscopy, and ophthalmoscopy were performed in each patient. A-scan ophthalmic ultrasonography and UBM were performed by the same experienced technician.

Rebound tonometry (TAO11, Icare Finland Oy, Finland): IOP measurements were performed thrice in each patient using a rebound tonometer, and the values were averaged.

A-scan ophthalmic ultrasonography (Quantel Medical, France): patients were placed in the supine position and, were asked to look at the finger directly above them, after the application of a topical anesthetic. Then, the lens thickness (LT) and AL of each patient were measured separately, with each parameter measured ten times and averaged.

UBM (3200L, Tianjin Suowei; probe frequency, 50 MHz): Patients were placed in the supine position under natural light, and the examination was carried out after dripping topical anesthetic. Images of the anterior chamber and the anterior chamber angle in the superior, inferior, nasal, and temporal quadrants (corresponding to 12, 6, 3, and 9 o’clock, respectively, in the case of the right eye) were separately captured, and higher-quality images were retained. Anterior chamber depth (ACD), PD, and lens vault (LV) were measured using the in-built software of the system, and mean values in the horizontal and vertical directions were calculated. The trabecular-iris angle (TIA), angle opening distance (AOD_500_), iris-lens angle (ILA), and iris-lens contact distance (ILCD) were measured in the superior, inferior, nasal, and temporal quadrants. Average values for the four quadrants were obtained and used for calculating the corresponding lens position (LP), relative lens position (RLP), and LT/AL factor (LAF) values using the following formulae: LP = ACD + 1/2LT; RLP = LP/AL × 10 (10, 11); LAF = LT/AL.

### 2.3 Measurement parameters

**Figure 1:**
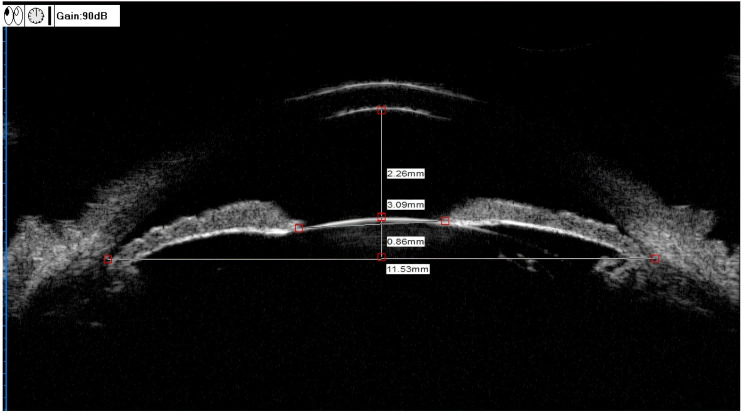
UBM measurements of the ACD, LV, and PD. ACD (anterior chamber depth) = 2.26 mm, LV (lens vault) = 0.86 mm, PD (pupil diameter) = 3.09 mm.

ACD (mm): the vertical distance from the inner surface of the central cornea to the anterior surface of the lens (**Figure 1**).

PD (mm): Distance between pupils measured on the iris cross-section (**Figure 1**).

LV (mm): The perpendicular distance from the anterior pole of the lens to the horizontal line between the scleral spurs (**Figure 1**).

**Figure 2:**
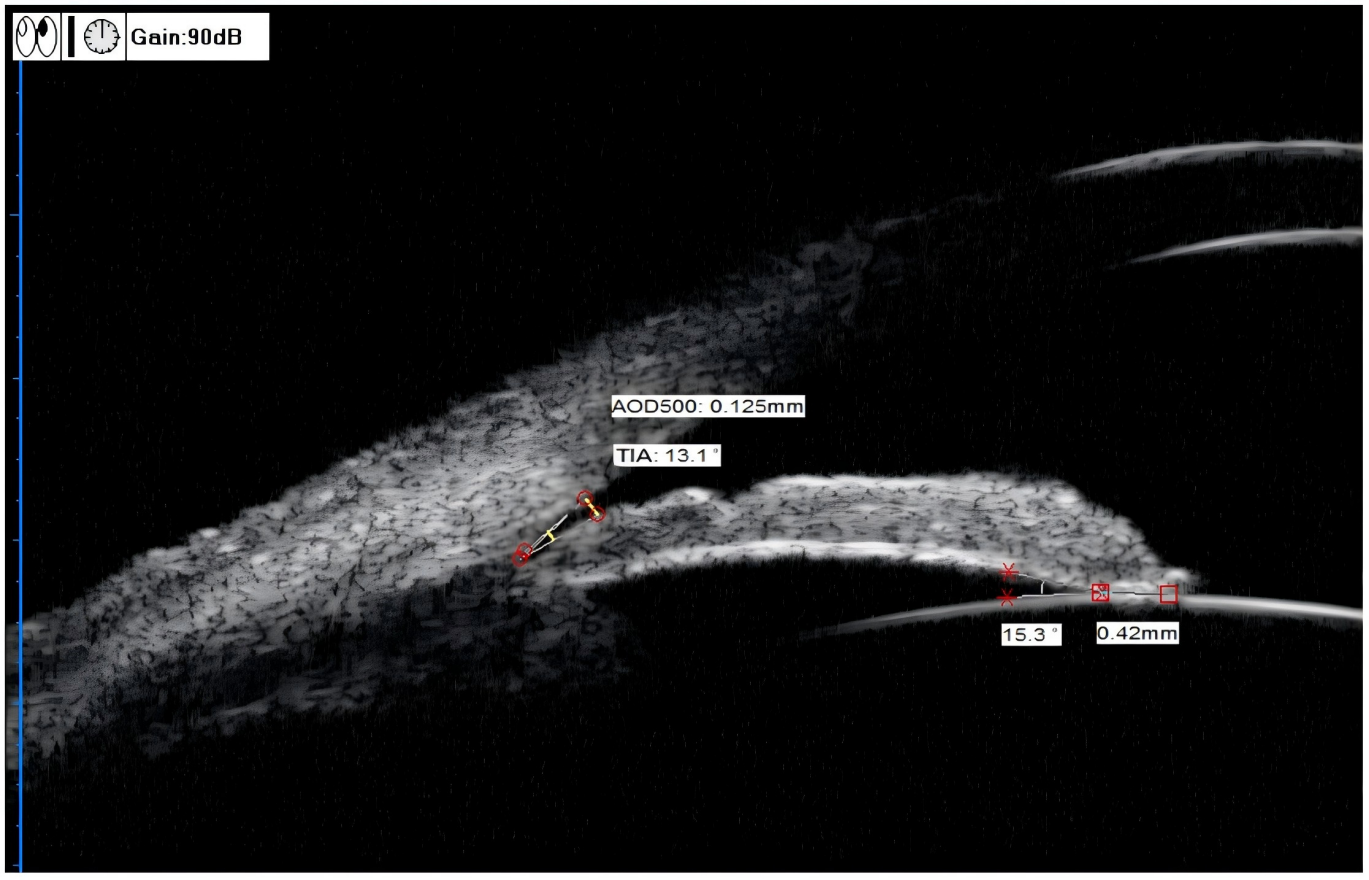
UBM measurements of the TIA, AOD_500_, ILA, and ILCD. TIA (trabecular-iris angle) = 13.1°, AOD_500_ (angle opening distance) = 0.125 mm, ILA(iris-lens angle), = 15.3°, ILCD (iris-lens contact distance) = 0.42 mm.

TIA (°): The clinical TIA value was consistent with the anterior chamber angle of 500 μm (anterior chamber angle at 500 μm from the scleral spur, TIA_500_). The specific measurement method was to make a triangle with AOD_500_ as the base and the recess at the iris root as the vertex, and the included angle of the vertex was TIA (**Figure 2**) (12).

AOD_500_ (mm): The specific measurement method was to start at a point 500 μm from the scleral spur along the corneal endothelium surface and make a line perpendicular to the corneal endothelium through this point. The perpendicular line intersected with the anterior iris surface. This vertical line was AOD_500_ (**Figure 2**) (13).

ILA (°): The specific measurement method was to take the contact point between the posterior iris surface and the anterior lens surface as the vertex, and two sides along this vertex were tangent lines of the posterior iris surface and the anterior lens surface, respectively. The included angle formed was ILA (**Figure 2**) (12).

ILCD (mm): The line between the contact points of the anterior and posterior iris surfaces and the anterior lens surface (**Figure 2**) (12).


LP = ACD + 1/2LT

RLP = LP/AL × 10

LAF = LT/AL

### 2.4 Statistical methods

The data were analyzed using IBM SPSS Statistics for Windows, version 27.0 (IBMCorp., Armonk, N.Y., USA). Normally distributed data were expressed as the mean ± standard deviation and compared between the two groups using an independent samples t-test. Data that did not follow a normal distribution were expressed as the M(*P*_25_, *P*_75_) and compared using the nonparametric Mann–Whitney U test. Differences were considered statistically significant when *P* < 0.05, and they were considered highly statistically significant when *P* < 0.01.

## 3 Results

### 3.1 Imaging analysis of anterior chamber angle-related parameters in APACG and CPACG

**Table 2** shows that the mean TIA, TIAmax-min, mean AOD_500_, and AOD_500 max-min_ of the APACG group were lower than those of the CPACG group, with the differences being statistically significant (*P* < 0.05).

**Table 2:** Comparison of anterior chamber-related parameters.

Parameters	APCAG (n=45)	CPCAG (n=37)	Mann-Whitney test	
	M (P_25_, P_75_)	M (P_25_, P_75_)	U	*P*
Mean TIA (°)	0.275 (0.000, 4.250)	3.000 (0.000, 17.650)	599.000	0.024
TIA_max-min_ (°)	1.100 (0.000, 11.250)	8.800 (0.000, 15.150)	627.500	0.048
Mean AOD_500_ (mm)	0.003 (0.000, 0.046)	0.038 (0.000, 0.203)	590.500	0.020
AOD_500 max-min_ (mm)	0.010 (0.000, 0.106)	0.111 (0.000, 0.198)	586.000	0.018

The Mann–Whitney U test was used for all data in the above table.

APACG, acute primary angle-closure glaucoma; CPACG, chronic primary angle-closure glaucoma; TIA, trabecular-iris angle; AOD500, angle opening distance.

### 3.2 Imaging analysis of lens position-related parameters in APACG and CPACG patients

**Figure 3:**
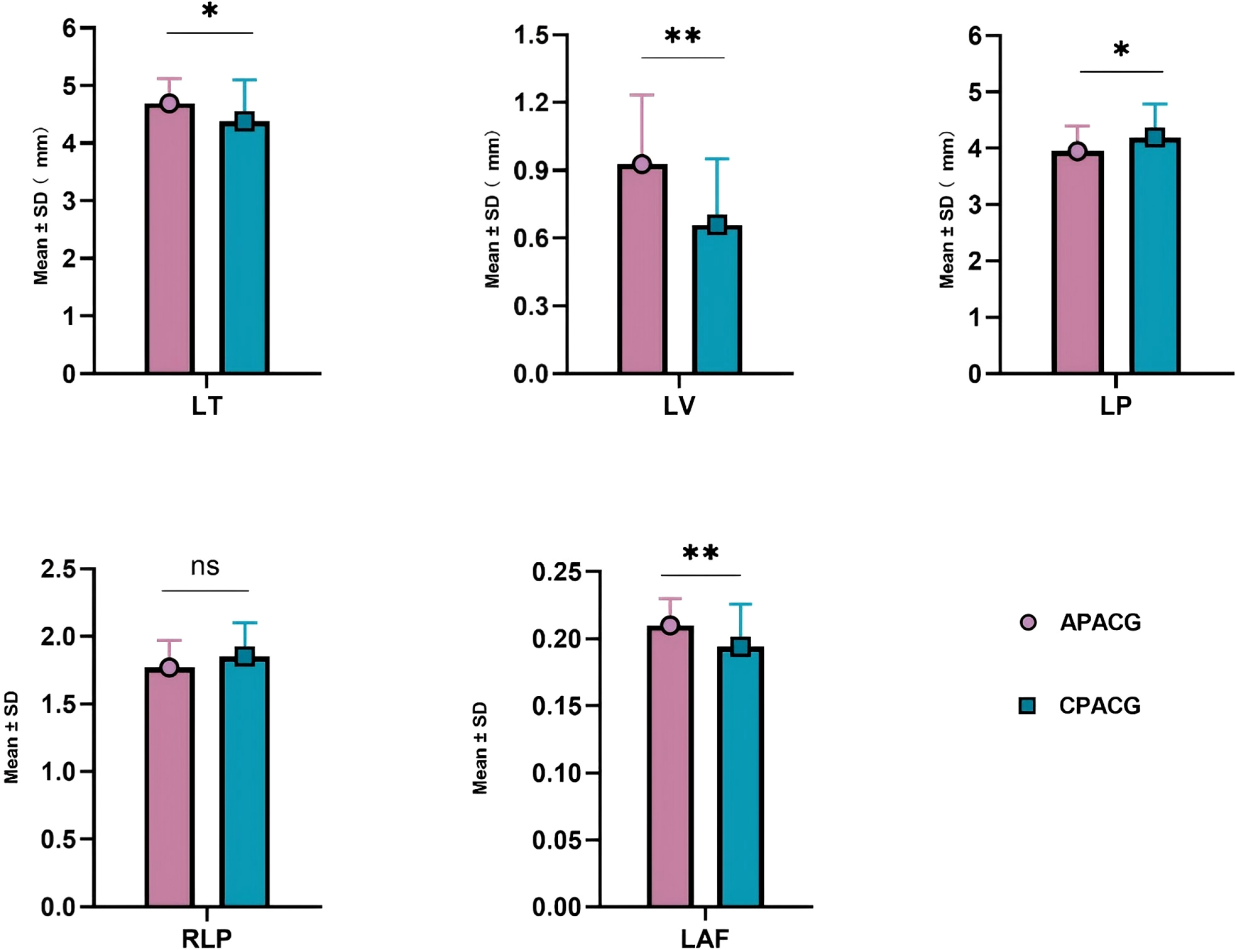
The comparison of lens position-related parameters. *P < 0.05; **P < 0.01; ns, not statistically significant. APACG, acute primary angle-closure glaucoma; CPACG, chronic primary angle-closure glaucoma; LT, lens thickness; LV, lens vault; LP, lens position; RLP, relative lens position; LAF, lens thickness/axial length factor.

**Table 3** shows that the ACD value of 1.603 ± 0.363 mm for the APACG group was significantly lower than the value of 2.000 ± 0.412 mm for the CPACG group, with the difference being highly statistically significant (*P* < 0.001). The APACG patients had a slightly shorter AL 22.318 ± 0.783 mm than the CPACG patients 22.632 ± 0.948 mm, with the difference being not statistically significant (*P* = 0.105). The mean LT 4.690 ± 0.430 mm was significantly higher than that of the CPACG patients 4.380 ± 0.717 mm, with the difference reaching statistical significance (*P* = 0.025). The LV of the APACG group 0.927 ± 0.307 mm was significantly larger than that of the CPACG group 0.659 ± 0.292 mm, with the difference being highly statistically significant (*P* < 0.001). The mean LP value of 3.947 ± 0.447 mm of the APACG patients was lower than the value of 4.190 ± 0.597 mm of the CPACG patients, with the difference being statistically significant (*P* = 0.038). The mean RLP value was also lower in the APACG group 1.770 ± 0.201 than in the CPACG group 1.851 ± 0.249, but the difference was not statistically significant (*P* = 0.104). The LAF of the APACG group 0.210 ± 0.020 was higher than that of the CPACG group 0.194 ± 0.032, with the difference being statistically significant (*P* = 0.009). And these comparisons were represented figuratively in the **Figure 3**.

**Table 3:** Comparison of lens position-related parameters.

Parameters	APCAG (n=45)	CPCAG (n=37)	Levene test		T-test	
	Mean ± SD	Mean ± SD	F	*P*	T	*P*
ACD (mm)	1.603 ± 0.363	2.000 ± 0.412	0.789	0.377	-4.648	<0.001^*^
AL (mm)	22.318 ± 0.783	22.632 ± 0.948	0.051	0.822	-1.642	0.105
LT (mm)	4.690 ± 0.430	4.380 ± 0.717	7.363	0.008	2.306	0.025
LV (mm)	0.927 ± 0.307	0.659 ± 0.292	1.125	0.292	4.021	<0.001^*^
LP (mm)	3.947 ± 0.447	4.190 ± 0.597	3.614	0.061	-2.107	0.038
RLP	1.770 ± 0.201	1.851 ± 0.249	1.342	0.250	-1.644	0.104
LAF	0.210 ± 0.020	0.194 ± 0.032	7.749	0.007	2.715	0.009^*^

*P < 0.01; An independent samples t-test was used for all data in the above table.

APACG, acute primary angle-closure glaucoma; CPACG, chronic primary angle-closure glaucoma; ACD, anterior chamber depth; AL, axial length; LT, lens thickness; LV, lens vault; LP, lens position; RLP, relative lens position; LAF, lens thickness/axial length factor.

The LP and RLP values of the APACG group were also lower than those of the CPACG group, but only the difference in LP values being statistically significant (*P* < 0.05). The LT, LV, and LAF values were higher than those of the CPACG group, with the differences reaching statistical significance (separately *P* < 0.05, *P* < 0.01, *P* < 0.01).

### 3.3 Imaging analysis of lens stability in APACG and CPACG patients

**Figure 4:**
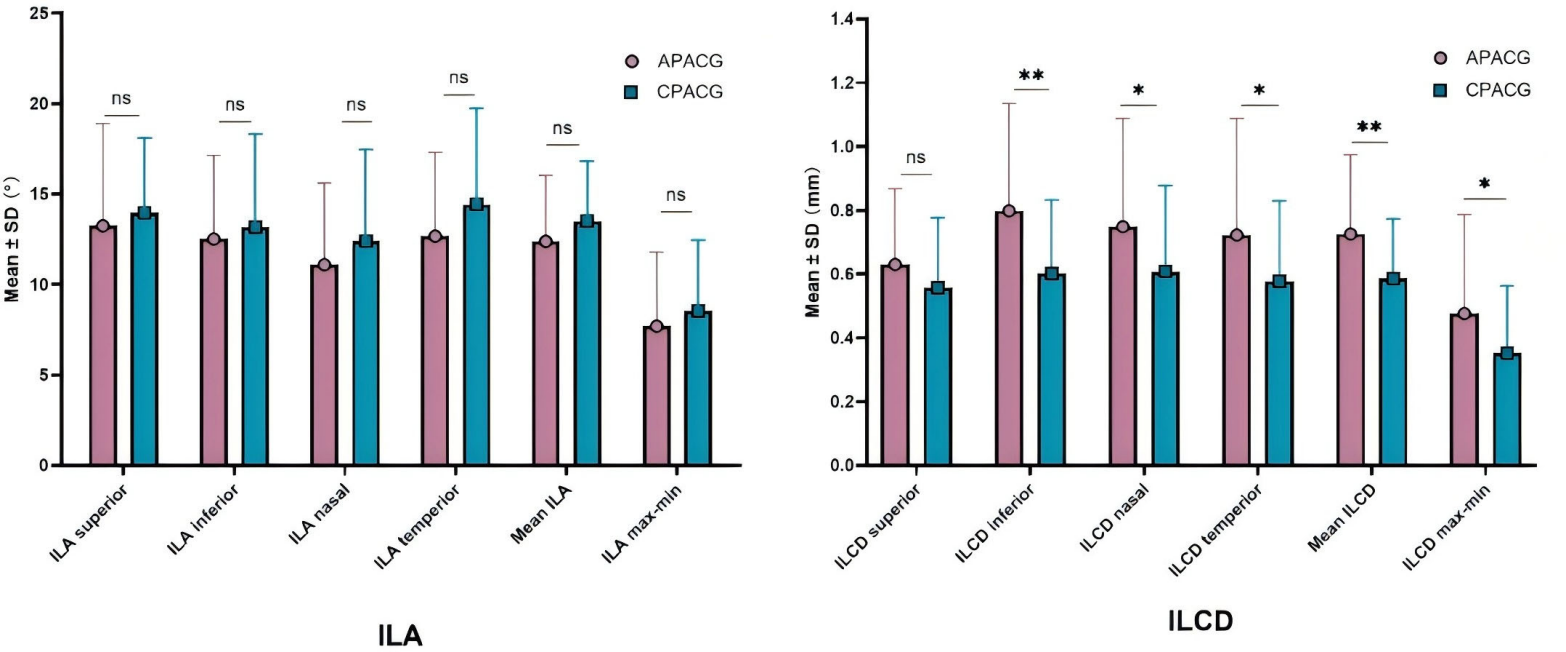
The comparison of lens stability-related parameters. *P < 0.05; **P < 0.01; ns: not statistically significant. APACG, acute primary angle-closure glaucoma; CPACG, chronic primary angle-closure glaucoma; ILA, iris-lens angle; ILCD, iris-lens contact distance.

**Table 4** shows that the mean ILA value of 12.373° ± 3.652° and ILA_max-min_ value of 7.693° ± 4.107° of the APACG patients were respectively slightly lower than the mean ILA value of 13.489° ± 3.350° and ILA_max-min_ value of 8.522° ± 3.926° of the CPACG patients, but the differences were not statistically significant (*P* = 0.157 and *P* = 0.357, respectively). The mean ILCD 0.725 ± 0.249 mm and ILCD_max-min_ 0.476 ± 0.311 mm of the APACG group were significantly larger than the corresponding values for the CPACG group 0.586 ± 0.187 mm and 0.352 ± 0.211 mm, respectively, with the differences reaching statistical significance (*P* < 0.05). And these comparisons were represented figuratively in the **Figure 4**.

**Table 4:** Comparison of lens stability-related parameters.

Parameters	APCAG (n=45)	CPCAG (n=37)	Levene test		T-test	
	Mean ± SD	Mean ± SD	F	*P*	T	*P*
ILA_superior_ (°)	13.236 ± 5.674	13.962 ± 4.132	2.241	0.138	-0.650	0.518
ILA_inferior_ (°)	12.500 ± 4.642	13.170 ± 5.158	0.095	0.758	-0.619	0.538
ILA_nasal_ (°)	11.091 ± 4. 513	12.397 ± 5.092	1.483	0.227	-1.231	0.222
ILA_temperior_(°)	12.667 ± 4.634	14.424 ± 5.288	0.416	0.521	-1.604	0.113
Mean ILA (°)	12.373 ± 3.652	13.489 ± 3.350	0.854	0.358	-1.428	0.157
ILA_max-min_ (°)	7.693 ± 4.107	8.522 ± 3.926	0.144	0.706	-0.927	0.357
ILCD_superior_ (mm)	0.630 ± 0.238	0.557 ± 0.219	0.178	0.674	1.448	0.152
ILCD_inferior_ (mm)	0.798 ± 0.337	0.602 ± 0.230	5.088	0.027	3.114	0.003^*^
ILCD_nasal_ (mm)	0.748 ± 0.341	0.608 ± 0.269	1.788	0.185	2.021	0.047
ILCD_temperior_ (mm)	0.722 ± 0.367	0.577 ± 0.253	4.626	0.035	2.119	0.037
Mean ILCD (mm)	0.725 ± 0.249	0.586 ± 0.187	4.015	0.048	2.875	0.005^*^
ILCD_max-min_ (mm)	0.476 ± 0.311	0.352 ± 0.211	4.334	0.041	2.139	0.036

*P* < 0.01. An independent samples t-test was used for all data in the above table.

APACG, acute primary angle-closure glaucoma; CPACG, chronic primary angle-closure glaucoma; ILA, iris-lens angle; ILCD, iris-lens contact distance.

The differences of ILA values in each quadrant, mean ILA, ILA_max-min_ between two groups showed no statistical significant (all *P* > 0.05).The ILCD values in each quadrant, mean ILCD, ILCD_max-min_ were higher in the APACG group than in the CPACG group, with the differences being more prominent in the inferior, nasal, and temporal quadrants, mean ILCD, and ILCD_max-min_ (separately *P* < 0.01, *P* < 0.05, *P* < 0.05, *P* < 0.01, *P* < 0.05).

## 4 Discussion

PACG is a common and important form of glaucoma. It can be categorized as APACG and CPACG. Lens-related factors are among the most important factors of PACG pathogenesis in aging individuals and have constantly received substantial research attention. Moghimi et al. identified a significant correlation between angle closure and an increase in LV (14). A study by Nongpiur et al. investigated the relationships between lens-related parameters (LV, LT, and LP) and angle closure and demonstrated that the LV was a new parameter other than age, gender, ACD and LT that was independently associated with angle closure (15). However, another study suggested an absence of significant correlations between RLP and glaucoma (16). Anatomical structure, pathogenesis, and disease progression are known to differ between these two forms of glaucoma, but it remains unclear whether differences in lens-related parameters, particularly lens position and stability, are manifested between eyes affected by APACG and CPACG. In the present study, we performed a comparative analysis of parameters related to lens position and stability in APACG patients and CPACG patients. Differences in parameters such as the LT, LV, ILA, ILCD, LP, RLP, and LAF were observed to provide a basis for the formulation of treatment regimens for these two forms of glaucoma.

It is known that the gradual increase in LT with age has a greater impact on PACG patients with anatomically narrow angles. Studies comparing the LT between glaucoma patients and normal participants have demonstrated that the LT was larger in PACG patients (17, 18). Liu et al. performed measurements of LT by AS-OCT and reported that the LT of APACG patients was 0.14 mm thicker than that of CPACG patients (19). Our results demonstrated that the LT was significantly larger and the ACD was significantly shallower in the APACG group than in the CPACG group. This corroborates the viewpoint that lens protrusion mainly occurs towards the anterior chamber, thus causing a decrease in anterior chamber depth (20). It is therefore evident that an increased LT is a risk factor for acute attacks of glaucoma.

LV reflects the degree of lens protrusion into the anterior chamber and also serves as an indicator of lens position. It has been demonstrated that the LV is closely associated with angle closure, with a greater degree of anterior lens protrusion possibly leading to a narrower anterior chamber angle and being more likely to induce progressive angle closure (14). Tian used UBM to compare biological parameters between APACG and CPACG patients (21). The results indicated that the APACG group had a significantly higher LV than the CPACG group. Liu et al. performed AS-OCT to examine 103 eyes from 81 patients (22 eyes with APACG, 22 eyes with preclinical APACG, and 59 eyes with cataract) (22). It was demonstrated that the APACG group had the largest LV, followed by the preclinical APACG and cataract groups. However, the difference in the LV between the APACG eyes and the contralateral eyes with preclinical APACG was not statistically significant (*P* = 0.659). The ILA and ILCD reflect the contact between the anterior lens surface and posterior iris surface and are correlated with the degree of LV protrusion. Previous research has demonstrated a significant negative correlation between these two parameters (23). Our findings indicate that both the LV and mean ILCD were significantly higher in the APACG group than in the CPACG group. The mean ILA of the APACG group was slightly lower than that of the CPACG group, but the difference was not significant. The mean TIA, TIA_max-min_, mean AOD_500_, and AOD_500 max-min_ are direct indicators of anterior chamber angle parameters. We demonstrated that the mean TIA, TIA_max-min_, mean AOD_500_, and AOD_500 max-min_ of the APACG group were significantly lower than those of the CPACG group. Therefore, it is evident that a higher LV caused greater anterior protrusion of the iris. This led to increased iris-lens contact, a higher ILCD, and a lower ILA. The resultant elongation and narrowing of the iris-lens channel increased the resistance to aqueous humor outflow, which induced pupillary block and ultimately triggered the acute attack of glaucoma (24). Liang et al. also concluded that the LV is an important structural indicator for distinguishing between APACG and CPACG (25).

The LP reflects the position of the lens-iris septum, and the RLP reflects the position of the lens relative to the overall ocular structure. Both parameters are indicative of the influence that the anatomical position of the lens has on the anterior chamber structure (26). Zhang et al. compared the lens parameters of APACG patients, CPACG patients, and normal participants using UBM and concluded that the RLP values of the three groups were significantly different (*P* < 0.05) (27).Compared with the CPACG group, the APACG group had a more-anterior RLP and shallower ACD, with the differences being statistically significant. Lim et al. measured anterior segment parameters in both the affected and contralateral eyes of APACG patients and compared the ACD, LP, and RLP values (28). It was demonstrated that the LP was more anterior in the affected eye than in the contralateral eye, whereas the RLP exhibited no significant difference between the two groups. We demonstrated that the LP value of the APACG patients was significantly lower than that of the CPACG group. The RLP value was also lower than that of the CPACG group, but the difference was not statistically significant. This suggests that both the lens-iris septum and lens were positioned more anteriorly in the APACG patients than in the CPACG patients.

The LAF also reflects the lens position and represents the proportion of the lens in the entire optical axis. A higher LAF is indicative of a thicker lens within the entire eye, which exerts a greater forward thrust on the iris and causes a higher tendency for angle closure. Li et al. compared the biological parameters of the lens between PACG patients, primary open-angle glaucoma (POAG) patients, and normal participants using AS-OCT (29). The results revealed that the LAF values of the POAG patients and normal participants did not differ significantly but were both significantly lower than that of the PACG patients. It was also demonstrated that the LAF of the APACG patients was higher than that of the CPACG patients. Wang et al. also demonstrated that the LAF of PACG-affected eyes was significantly higher than that of normal eyes (30). Similarly, our findings demonstrated that the LAF was significantly higher in the APACG group than in the CPACG group, with the difference being statistically significant. It is known that the LT is negatively correlated with the AL, whereas the RLP is positively correlated with the AL in a normal eye. Compared with normal eyes, eyes with a longer AL have a relatively thinner lens, lower LAF, and a more-posterior RLP, whereas eyes with a shorter AL have a relatively thicker lens, higher LAF, and a more-anterior RLP (26).

Slackening of the suspensory ligament with increasing age may cause displacement or deviation of the lens, which often move forward. Therefore, the laxity of the suspensory ligament is a key factor affecting lens stability. The ILA_max-min_ and ILCD_max-min_ values of the various quadrants are important indicators for lens stability (23). Although the differences of ILA values in the superior, inferior, nasal, and temporal quadrants between two groups showed no statistical difference, the every quadrant of the APACG group was lower than the corresponding values of the CPACG group. The ILCD values in the four quadrants were higher in the APACG group than in the CPACG group, with the differences being more prominent in the inferior, nasal, and temporal quadrants. In addition, the ILCD_max-min_ values in the four quadrants of the APACG group were significantly higher than that of the CPACG group. This quadrant change indicates nonuniform laxity of the suspensory ligament of the lens across the various quadrants of the APACG group, which suggested a poor lens stability and greater tendency for anterior displacement or even deviation. Whether it was the “cause” or “effect” of lens instability and the acute attack of APACG, which was still unknown and may be mutually causal, requiring further research.

This study has certain limitations. First, the relatively small sample size may have led to the lack of statistical significance in certain parameters despite the presence of differences between the two groups. Future studies involving large sample sizes will be required for validation of our findings. Second, the occurrence of partial recovery of certain signs, such as IOP, in APACG patients after undergoing pharmacological treatment was not entirely representative of the actual patient status during the acute attacks of glaucoma. Third, the APACG eyes included in this study had poor drug control for acute attacks, with only partial IOP control and needing surgeries, excluding those who had good IOP control with drug and/or laser therapy. For better comparability between the two groups, the enrolled CPACG eyes were also those with poor drug IOP control and continuous progression of optic nerve damage. The results were not comprehensive, and we may need to consider expanding such samples in the future.

## 5 Conclusion

In conclusion, the UBM imaging results indicated that APACG eyes had a thicker lens, more-anterior RLP, shallower anterior chamber, and greater susceptibility to pupillary block and angle closure than eyes with CPACG. The APACG group also exhibited nonuniform laxity in the suspensory ligament of the lens across the various quadrants. This contributed to poor lens stability and a greater tendency for anterior displacement or deviation of the lens. Therefore, greater attention should be paid to lens-related factors in APACG patients, and lens position and stability should be taken into consideration during the selection of treatment regimens.

## Data Availability

The raw data supporting the conclusions of this article will be made available by the authors, without undue reservation.
